# Supplementary material for: Comprehensive Evaluation of Multiple Approaches Targeting ABCB1 to Resensitize Docetaxel-Resistant Prostate Cancer Cell Lines
Source: Int J Mol Sci. 2022 Dec 30;24(1):666. doi: 10.3390/ijms24010666 (PMC9820728; doi:10.3390/ijms24010666)
Supplement: Supplementary file 1 [file ijms-24-00666-s001.zip › ijms-2027029-supplementary.pdf]

Supplementary information

Determination of the optimal treatment dose for ABCB1 inhibitors

For the evaluation of ABCB1 inhibitors, cells were seeded in triplicates in 96-well plates and cultured to ~50% confluence. To determine the highest dose of each ABCB1 inhibitor, that did not affect cell viability (maximum non-toxic dose, MNTD), DU145 and PC-3 CTRL and DTXR cells were treated with a serial dilution of either elacridar or tariquidar (1 nM – 100 μM) for 24 h. Since both inhibitors were solved in dimethyl sulfoxide (DMSO), the highest DMSO concentration (1%) was also investigated. To determine the minimal effective dose (MED) of elacridar and tariquidar for resensitization to DTX, DU145 and PC-3 DTXR cells were treated with a serial dilution of either elacridar or tariquidar (0,5 nM – 5 μM) in combination with 20 nM DTX for 24 h. Thereafter, the treatment was substituted by fresh cell culture medium and 24 h later, a WST-1 assay was performed.

A concentration of 1 μM was determined as the MNTD for each ABCB1 inhibitor in the four investigated cell lines (Suppl. Fig. S1a). ABCB1 inhibitor concentrations above 10 μM markedly impaired the cellular viability, whereupon the extent depended on the cell line. Since the inhibitors were solved in DMSO, a treatment with 1% DMSO was also performed, which corresponds to a treatment with 100 μM of the inhibitors. As seen in Suppl. Fig. S1a, 1% DMSO had no or only a small effect on the metabolic activity of the cells.

Next, the MED of both inhibitors, which was defined as their lowest concentration with effective resensitization to DTX in DTXR cells, was determined. In combination with 20 nM DTX, treatment with 50 nM of either elacridar or tariquidar highly diminished cellular viability of DU145 and PC-3 DTXR cells by about 35-75% (Suppl. Fig. S1b). Of note, elacridar was slightly more effective in the resensitization of DTXR cells than tariquidar. Consequently, a concentration of 50 nM elacridar or tariquidar was chosen for subsequent experiments. As shown above, the treatment with either 50 nM elacridar or tariquidar led to a reduction of the metabolic activity of less than 10% compared to the untreated cells (Suppl. Fig. S1a).

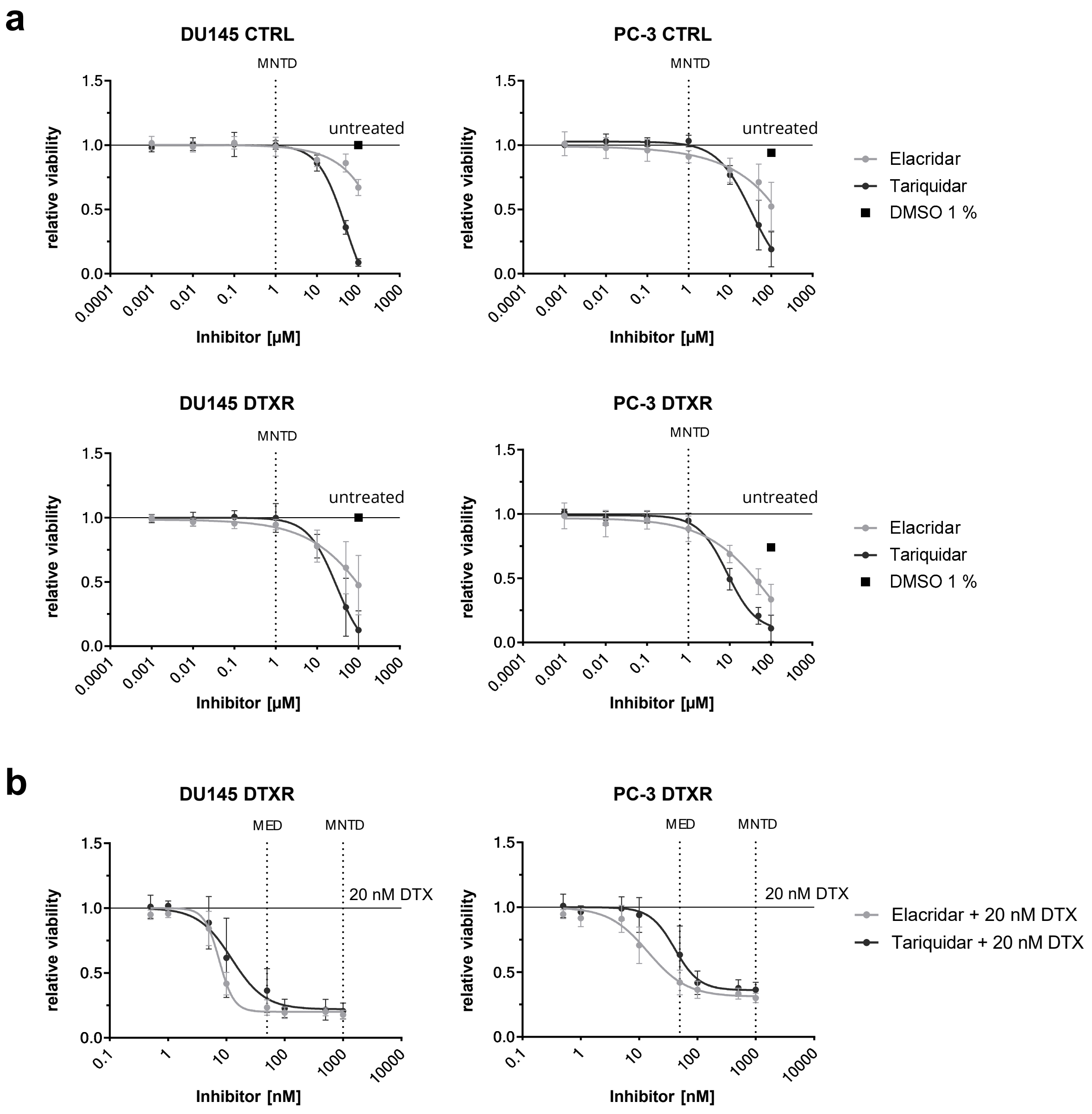

Suppl. Figure S1. Influence of the ABCB1 inhibitors elacridar and tariquidar alone or in combination with DTX on the cellular viability of DU145 and PC-3 cells. (**a**) DU145 and PC-3 CTRL and DTXR cells were treated with a serial dilution of the ABCB1 inhibitors elacridar and tariquidar as well as with the highest concentration of the solvent DMSO (1%). After treatment, the metabolic activity was measured by the WST-1 assay. MNTD: maximum non-toxic dose. (**b**) DU145 and PC-3 DTXR cells were treated with 20 nM DTX alone or in combination with a serial dilution of the ABCB1 inhibitors elacridar and tariquidar. After treatment, the metabolic activity was measured by the WST-1 assay. MED: minimal effective dose, MNTD: maximum non-toxic dose.

All data were normalized to the indicated control (black line). Data are depicted as mean ± SD of three to four independent biological replicates.

Results for the crystal violet assays

The respective results are mentioned in the main text (Sections 2.2. & 2.3.).

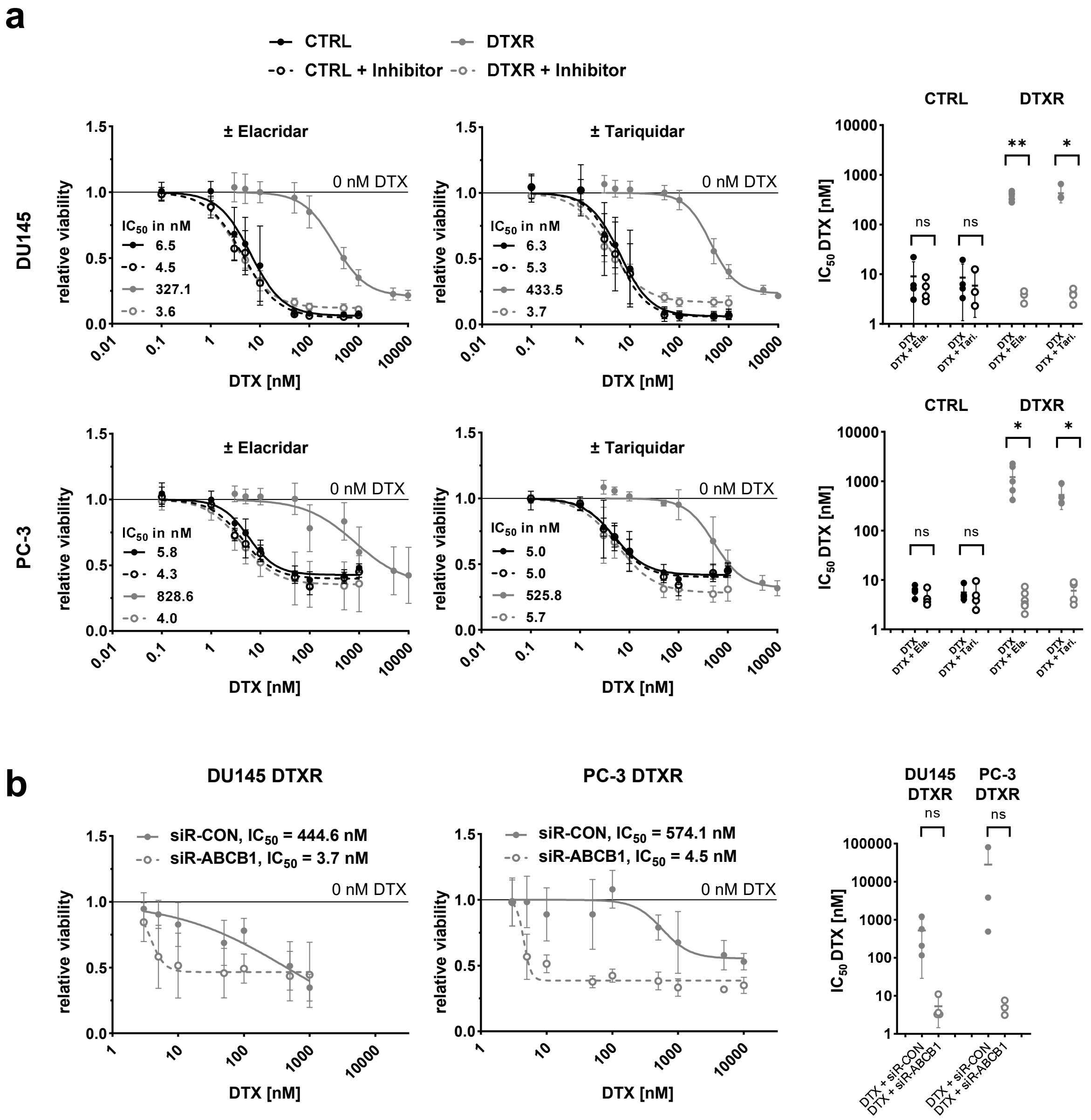

Suppl. Figure S2. Influence of the ABCB1 inhibitors elacridar and tariquidar and siRNA-mediated knockdown of *ABCB1* in combination with DTX on the cellular viability of DU145 and PC-3 DTXR cells compared to CTRL cells. (**a**) DU145 and PC-3 CTRL and DTXR cells were treated with a serial dilution of DTX in the presence or absence of an ABCB1 inhibitor (50 nM elacridar or tariquidar). After treatment, total adherent cell mass was determined by the crystal violet assay and IC_{50} values for DTX were calculated. The adjacent graphs to the right depict IC_{50} values calculated from individual experiments. Ela.: elacridar, Tari.: tariquidar. (**b**) DU145 and PC-3 DTXR cells were treated with a serial dilution of DTX after transfection with either siR-CON or siR-ABCB1 (20 nM). After treatment, total adherent cell mass was determined by the crystal violet assay and IC_{50} values for DTX were calculated. The adjacent graphs to the right depict IC_{50} values calculated from individual experiments.

All data were normalized to the indicated control (black line). Data are depicted as mean ± SD of three to six independent biological replicates. Non-paired t-test with Welch's correction for comparison of two treatment groups: * $p < 0.05$, ** $p < 0.01$, ns: not significant.

Densitometrical analysis of western blots determining parts of the total ABCB1 protein amount

For the analysis and comparability of the glycosylation status of ABCB1 protein after different treatments, western blots were analyzed densitometrically in order to obtain the amount of fully glycosylated, core-glycosylated and unglycosylated protein as part of the total ABCB1 protein amount in %. Following densitometrical analysis using ImageJ (Suppl. Fig. S3a), plots were generated that depict protein band intensities and thus, correlate with the amount of total protein (Suppl. Fig. S3b). These plots were manually separated into three parts and separation lines were positioned at the lowest point between two peaks indicating the space (brighter background) between to bands in the western blot image. For a better comparability, the separation lines were set at the same position for all treatment conditions. The area of each separated part was normalized to the respective reference protein (GAPDH or α-tubulin) and subsequently the percentage of each glycosylation status was calculated with respect to the total ABCB1 amount, which was computed by summation of the three individual parts (Suppl. Table S1).

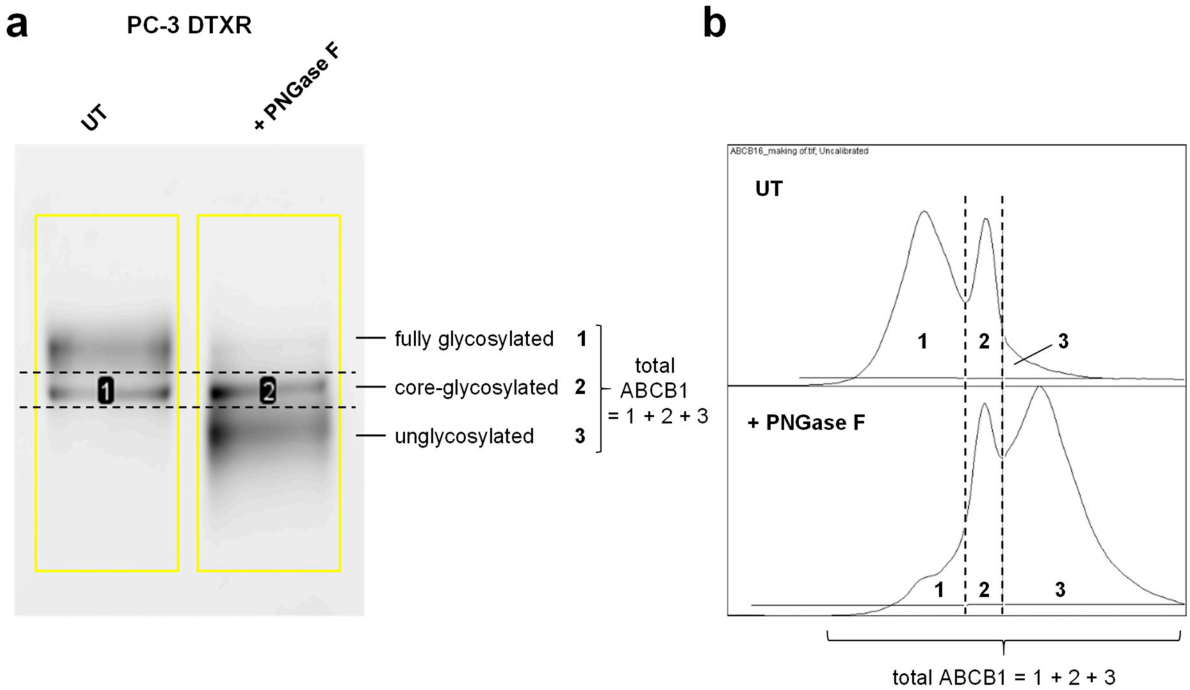

Suppl. Figure S3: Method of the densitometrical analysis of western blots in order to determine parts of the total ABCB1 protein amount in %, exemplarily shown for PC-3 DTXR cells with or without PNGase F digestion.

Suppl. Table S1. Exemplary densitometrical analysis of ABCB1 protein bands of PC-3 DTXR cells with or without PNGase F digestion. The area for each glycosylation status - fully glycosylated (fully glyc.), core-glycosylated (core-glyc.) and unglycosylated (unglyc.) - was normalized to the area of the reference protein GAPDH. Finally, parts of the total ABCB1 amount in % were calculated.

	ABCB1				GAPDH	ABCB1/GAPDH			
	**fully glyc.**	**core-glyc.**	**unglyc.**	**total**	**total**	**fully glyc.**	**core-glyc.**	**unglyc.**	**total**
**UT**	19662.9	8400.2	2213.9	30277.0	5065.9	3.88	1.66	0.44	5.98
**PNGase F**	4297.6	12064.6	33794.2	50156.4	6552.2	0.66	1.84	5.16	7.65
**part of total ABCB1 protein amount in %**						64.9	27.8	7.3	100
						8.6	24.0	67.4	100

Determination of the optimal treatment dose for tunicamycin

The optimal concentration of tunicamycin was determined by dose-response experiments and western blot analysis. For dose-response experiments, the DU145 and PC-3 DTXR cells were seeded in triplicates in 96-well plates as described and then pre-treated with a serial dilution of tunicamycin (5 – 750 ng/mL). After 96 h, a WST-1 assay was performed. For western blot analysis, the DU145 and PC-3 DTXR cells were seeded and pre-treated with tunicamycin (50 & 100 ng/mL) in 10 cm dishes and after 96 h of incubation, proteins were extracted and analyzed as described.

In DU145 and PC-3 DTXR cells, tunicamycin concentrations above 150 ng/mL markedly impaired the cellular viability after pre-treatment for 96 h (Suppl. Fig. S4a). In contrast, a tunicamycin concentration of 100 ng/mL reduced the metabolic activity only by about 10% in PC-3 DTXR cells compared to the untreated control cells and had no negative effect on the metabolic activity of DU145 DTXR cells.

Next, 50 and 100 ng/mL tunicamycin were investigated regarding their effect on the glycosylation status of the ABCB1 protein. However, only pre-treatment with 100 ng/mL tunicamycin resulted in a noticeable change in glycosylation status of the ABCB1 protein (Suppl. Fig. S4b). Therefore, 100 ng/mL tunicamycin was chosen for subsequent experiments since that concentration was effective in deglycosylation of ABCB1 protein, while keeping cell toxicity at a tolerable level.

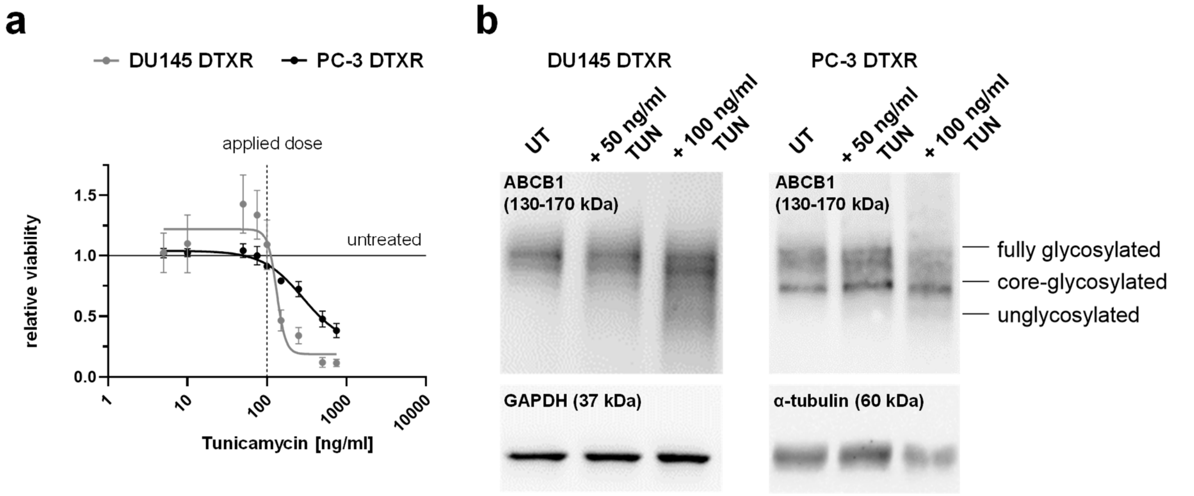

Suppl. Figure S4. Influence of tunicamycin treatment on the cellular viability and glycosylation status of ABCB1 protein in DU145 and PC-3 DTXR cells. (**a**) DU145 and PC-3 DTXR cells were pre-treated with a serial dilution of tunicamycin for 96 h. After treatment, the metabolic activity was measured by the WST-1 assay. The applied dose for subsequent experiments is indicated. All data were normalized to the indicated control (black line). Data are depicted as mean ± SD of three to four independent biological replicates. (**b**) DU145 and PC-3 DTXR cells were pre-treated with 50 or 100 ng/mL tunicamycin (TUN) for 96 h and compared to untreated cells (UT). Exemplary western blots for the detection of ABCB1 protein with GAPDH or α-tubulin as reference protein are shown.

Influence of tunicamycin on the total amount of ABCB1 protein

The total ABCB1 protein amount was calculated from the different protein bands representing fully, core- and unglycolysated ABCB1 protein of DTXR cells with and without tunicamycin pre-treatment (for explanation see Suppl. Fig. S3). The respective results (Suppl. Fig. S5a) are mentioned in the main text (Section 2.4.).

In order to confirm the absence of proteasomal degradation following deglycosylation, DU145 and PC-3 DTXR cells were treated with a combination of tunicamycin and the proteasome inhibitor MG132. For western blot analysis, DU145 and PC-3 DTXR cells were seeded in 10 cm dishes and cultured to ~50% confluence. According to a protocol by Nakagawa et al. [40], the cells were then pre-treated with 1 μM MG132 for 1 h and subsequently 100 μg/mL tunicamycin was added. After 96 h of incubation, proteins were extracted and analyzed as described. The respective results (Suppl. Fig. S5b) are mentioned in the main text (Section 2.4.).

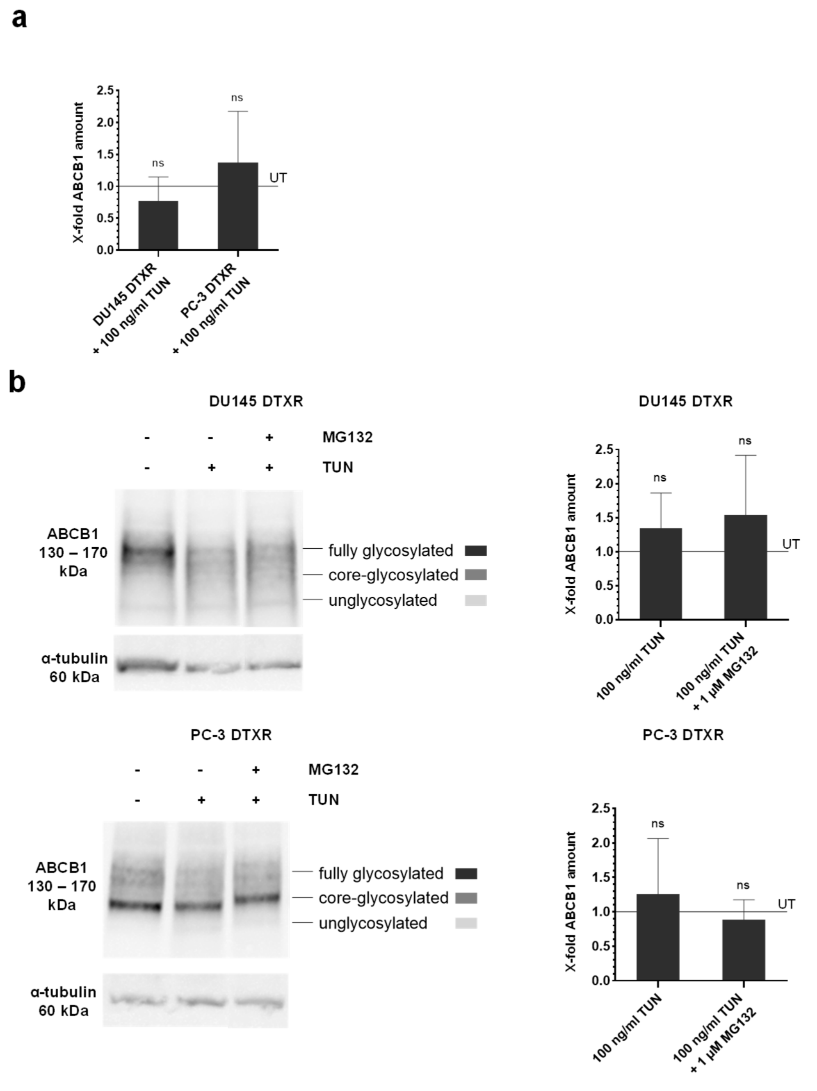

Suppl. Figure S5. Influence of tunicamycin treatment on the total amount of ABCB1 protein in DU145 and PC-3 DTXR cells. DU145 and PC-3 DTXR cells were pre-treated with 100 ng/mL tunicamycin (TUN) for 96 h (**a**) alone or (**b**) in combination with the proteasome inhibitor MG132. For the combinatory treatment, an exemplary western blot for detecting ABCB1 protein with α-tubulin as reference protein is shown. The protein bands were analyzed densitometrically, normalized to the respective reference protein and then the total amount of ABCB1 protein compared to untreated cells (UT; black line) was determined.

Data are depicted as mean ± SD of two to five independent biological replicates. One-sample t-test for comparison to the untreated controls: ns = not significant
